# Prediction of Occurrence of Cerebral Infarction After Successful Mechanical Thrombectomy for Ischemic Stroke in the Anterior Circulation by Arterial Spin Labeling

**DOI:** 10.1007/s00062-023-01295-x

**Published:** 2023-06-06

**Authors:** Masamune Kidoguchi, Ayumi Akazawa, Osamu Komori, Makoto Isozaki, Yoshifumi Higashino, Satoshi Kawajiri, Shinsuke Yamada, Toshiaki Kodera, Hidetaka Arishima, Tetsuya Tsujikawa, Hirohiko Kimura, Kenichiro Kikuta

**Affiliations:** 1 Department of Neurosurgery, Division of Medicine, Faculty of Medical Sciences, University of Fukui, 23–3 Matsuokashimoaizuki Eiheiji, 910-1193 Eiheiji-cho, Yoshida-gun, Fukui, Japan; 2 Department of Computer and Information Science, Faculty of Science and Technology, Seikei University, Musashino, Tokyo, Japan; 3 Department of Radiology, Division of Medicine Radiology and Laboratory Medicine, Faculty of Medical Sciences, University of Fukui, Eiheiji, Fukui, Japan

**Keywords:** Mechanical thrombectomy, Arterial spin labeling, ASPECTS, Outcome prediction, Diffusion tensor imaging, Ischemic penumbra

## Abstract

**Purpose:**

The overall goal of our study is to create modified Alberta Stroke Program Early Computed Tomography Score (ASPECTS) determined by the findings on arterial spin labeling imaging (ASL) to predict the prognosis of patients with acute ischemic stroke after successful mechanical thrombectomy (MT). Prior to that, we examined predictive factors including the value of cerebral blood flow (CBF) measured by ASL for occurrence of cerebral infarction at the region of interest (ROI) used in the ASPECTS after successful MT.

**Methods:**

Of the 92 consecutive patients with acute ischemic stroke treated with MT at our institution between April 2013 and April 2021, a total of 26 patients who arrived within 8 h after stroke onset and underwent MT resulting in a thrombolysis in cerebral infarction score of 2B or 3 were analyzed. Magnetic resonance imaging, including diffusion-weighted imaging (DWI) and ASL, was performed on arrival and the day after MT. The asymmetry index (AI) of CBF by ASL (ASL-CBF) before MT was calculated for 11 regions of interest using the DWI-Alberta Stroke Program Early CT Score.

**Results:**

Occurrence of infarction after successful MT for ischemic stroke in the anterior circulation can be expected when the formula 0.3211 × history of atrial fibrillation +0.0096 × the AI of ASL-CBF before MT (%) +0.0012 × the time from onset to reperfusion (min) yields a value below 1.0 or when the AI of ASL-CBF before MT is below 61.5%.

**Conclusion:**

The AI of ASL-CBF before MT or a combination of a history of atrial fibrillation, the AI of ASL-CBF before MT, and the time from onset to reperfusion can be used to predict the occurrence of infarction in patients arriving within 8 h after stroke onset in which reperfusion with MT was successful.

## Introduction

Several randomized clinical trials have demonstrated that mechanical thrombectomy (MT) combined with intravenous administration of tissue plasminogen activator (tPA) for patients in the early stage of acute ischemic stroke can result in better outcomes 90 days after the stroke, lower mortality rates, lower frequency of hemorrhagic complications, and an improved revascularization rate [1–5]. In addition to the time from onset to reperfusion, patient selection based on the evaluation of ischemic penumbra on arrival is another key to successful MT. Penumbra evaluation is performed using the National Institutes of Health Stroke Scale (NIHSS) [1, 6], multiphase computed tomography angiography (CTA) [2], with [5] or without [4] rapid processing of perfusion and diffusion (RAPID), perfusion CT [3], the Alberta Stroke Program Early Computed Tomography Score (ASPECTS) [2, 4, 5], and ASPECTS of diffusion-weighted imaging (DWI-ASPECTS) [2, 4, 5]. Arterial spin labeling (ASL) is one of the most noninvasive magnetic resonance imaging (MRI) methods for imaging cerebral blood flow (CBF) [7, 8]. As ASL does not require a contrast medium and provides images within a short time, it is suitable for the diagnosis and evaluation of cerebral blood flow (CBF) in acute stroke patients [9–12] and for the prediction of stroke prognosis [13–16]; however, few studies have examined the role of ASL in MT [17, 18]. The overall goal of our study is to create modified ASPECTS determined by the findings on ASL to predict the prognosis of the patient with acute ischemic stroke after successful MT. Prior to that we examined predicting factors including the value of CBF measured by ASL for occurrence of cerebral infarction at regions of interest (ROI) used in the ASPECTS after successful MT.

## Methods

### Eligibility Criteria

**Fig. 1:**
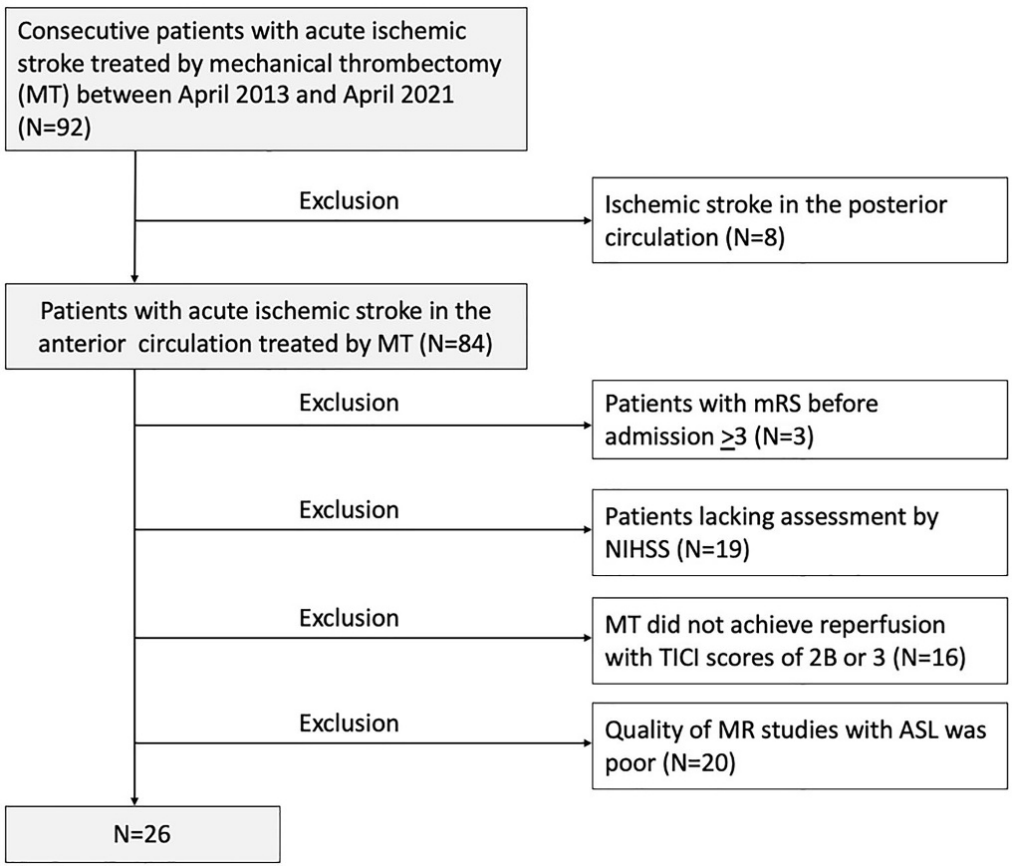
Inclusion criteria of patients in the study

Of the 92 consecutive patients with acute ischemic stroke treated with MT at our institution between April 2013 and April 2021, we initially excluded 8 patients with stroke in the posterior circulation. Thus, 84 patients with acute ischemic stroke in the anterior circulation treated with MT were eligible for this retrospective cohort study. Excluded were 3 patients with a modified Rankin Scale (mRS) score > 3 before admission, 19 patients lacking NIHSS assessment results, 16 patients in which reperfusion with MT did not result in a thrombolysis in cerebral infarction (TICI) score of 2B or 3 by MT, and 20 patients with poor ASL images. Finally, we analyzed 26 patients with ischemic stroke in the anterior circulation who arrived within 8 h after onset and underwent MT with excellent reperfusion. This study was approved by the ethics committee of the University of Fukui (No. 20170214), and informed consent was obtained from all patients (Fig. 1).

### MT Procedures

Onset time was defined as the time when ischemic symptoms occurred or the latest time when the patient was observed in good health. Patients who arrived at our institution within 8 h after onset were examined with CT and MRI for a diagnosis of acute ischemic stroke in the anterior circulation. Patients who arrived within 4.5 h after stroke onset were treated with intravenous alteplase (0.6 mg/kg body weight) according to the Japanese guidelines for the management of stroke [19, 20]. Those who arrived within 8 h after onset were treated with MT using contact aspiration and/or the stent retriever technique with a balloon-guiding catheter.

### Imaging Study

All patients underwent various types of MRI procedures with a 3 T unit (3.0 T; Discovery 750, version DV25.1; GE Healthcare, Waukesha, WI, USA) including DWI, MR angiography, and pulsed continuous ASL (repetition time = 4844 ms, echo time = 10.5 ms, slice thickness = 4 mm, post-labeling delay = 2025 ms, labeling duration = 1450 ms) on admission and the day after MT. The imaging time for ASL and total MRI procedure was 4 min 7 s and 15 min 55 s, respectively.

### Regions of Interest (ROI) for Assessment of CBF Values Calculated by ASL

We prepared 11 ROIs for each of the 26 patients to be used in DWI-ASPECTS for the assessment of CBF. In total, we derived CBF values (ASL-CBF) for 286 ROIs according to the MRI results obtained before MT. We calculated the asymmetry index (AI) of ASL-CBF as the ratio (%) of the ASL-CBF of a ROI on the affected side to that of a ROI on the contralateral side. The occurrence of CI in the 286 ROIs was determined using DWI after MT.

### Statistical Analysis

Univariate analysis was performed using Pearson’s χ^2^-test and Fisher’s exact test for categorical variables and the Mann-Whitney U‑test for numerical variables. Forward and backward stepwise logistic regression analyses with the Akaike Information Criterion (AIC) were carried out to determine the associations between the occurrence of CI after MT and factors such as age, sex, history of hypertension, hyperlipidemia, diabetes mellitus, current smoking habit, administration of alteplase before MT, duration from onset to MRI examination, duration from onset to reperfusion and CBF value before MT. The cut-off value for CBF from a receiver operating characteristic (ROC) analysis using the area under the curve (AUC) and logistic regression was calculated using Bantis’s method [21]. All statistical analyses were performed by a professional independent statistician (OK), using JMP 15.2.0 (SAS Institute, Cary, NC, USA) and R (R Foundation for Statistical Computing, Vienna, Austria), with a *p*-value < 0.05 indicating statistical significance.

## Results

### Patient Characteristics

There were 18 men and 8 women in the 26 patients in this study. The age ranged from 24 to 91 years (mean 67 ± 19 years). The occlusion site of the vessel was right internal carotid artery (ICA) in 1 patient, left ICA in 3 patients, right M1 portion of middle cerebral artery (MCA) in 5 patients, left M1 portion of MCA in 11 patients, right M2 portion of MCA in 3 patients, and left M2 portion of MCA in 2 patients. The mean mRS scores before admission were 0.12 ± 0.32 points and NIHSS scores on admission were 16.6 ± 7.6 points. Of the patients 11 had a history of hypertension, 6 had hyperlipidemia, 4 patients had diabetes mellitus, 13 had atrial fibrillation (AF), and 9 patients were current smokers. Furthermore, 13 patients received intravenous administration of alteplase before MT and 13 patients did not. The mean time from onset to reperfusion was 279 min. Additionally, reperfusion with a TICI score of 3 and of 2B was achieved in 13 patients and 13 patients, respectively. No patients developed symptomatic intracranial hemorrhage after MT (Table 1).

**Table 1:** Patient characteristics

Variable			
No. of cases			26
Age in years (mean ± SD)			24–91 (67 ± 19)
Sex	Male		18 (69%)
	Female		8 (31%)
Occlusion site of vessel	Internal carotid artery	Right	1 (4%)
		Left	3 (12%)
	M1 portion of middle cerebral artery	Right	5 (19%)
		Left	11 (42%)
	M2 portion of middle cerebral artery	Right	3 (12%)
		Left	2 (8%)
mRS scores before admission (points) (mean ± SD)			0–1 (0.12 ± 0.32)
NIHSS scores on admission (points) (mean ± SD)			3–30 (16.6 ± 7.6)
History of	Hypertension		11 (42%)
	Hyperlipidemia		6 (23%)
	Diabetes mellitus		4 (15%)
	Atrial fibrillation		13 (50%)
	Current smoking		9 (34%)
MT	With intravenous alteplase		13 (50%)
	Without intravenous alteplase		13 (50%)
Time from onset to reperfusion (min) (mean ± SD)			105–729 (279 ± 142)
TICI score at MT	2B		13 (50%)
	3		13 (50%)

*MT* mechanical thrombectomy, *TICI score* thrombolysis in cerebral infarction score

### Univariate Analysis of Factors Predicting CI in 286 ROIs After Successful MT

Of all investigated factors (age, sex, history of hypertension, hyperlipidemia, diabetes mellitus, or atrial fibrillation, current smoking habit, administration of alteplase before MT; AI of ASL-CBF before MT, time from onset to reperfusion), the AI of ASL-CBF before MT (*p* = 0.0005) was the only significant predictor of CI after successful reperfusion with MT (Table 2).

**Table 2:** Univariate analysis for factors predicting occurrence of cerebral infarction at 286 ROIs after successful reperfusion by MT

Variable		Occurrence of CI	No occurrence of CI	*p* value
Number of ROIs		97	189	–
Age (years, mean±SD)		67 ± 21	68 ± 17	0.8553
Male		68 (70%)	130 (69%)	0.8926
History of	Hypertension	39 (49%)	82 (43%)	0.6159
	Hyperlipidemia	22 (23%)	44 (23%)	1.0000
	Diabetes mellitus	12 (12%)	32 (17%)	0.3875
	Atrial fibrillation	46 (47%)	97 (51%)	0.6175
	Current smoking	31 (32%)	68 (36%)	0.5148
Administration of alteplase before MT		41 (43%)	102 (54%)	0.0801
AI of ASL-CBF before MT (%)		54 ± 27	64 ± 29	0.0005*
Time from onset to reperfusion (minutes)		266 ± 109	285 ± 152	0.9934

*ROI* region of interest, *MT* mechanical thrombectomy, *AI* asymmetry index, *ASL* arterial spin labeling, *CBF* cerebral blood flow

### Logistic Regression Analysis of Factors Predicting CI in 286 ROIs After Successful MT

**Fig. 2:**
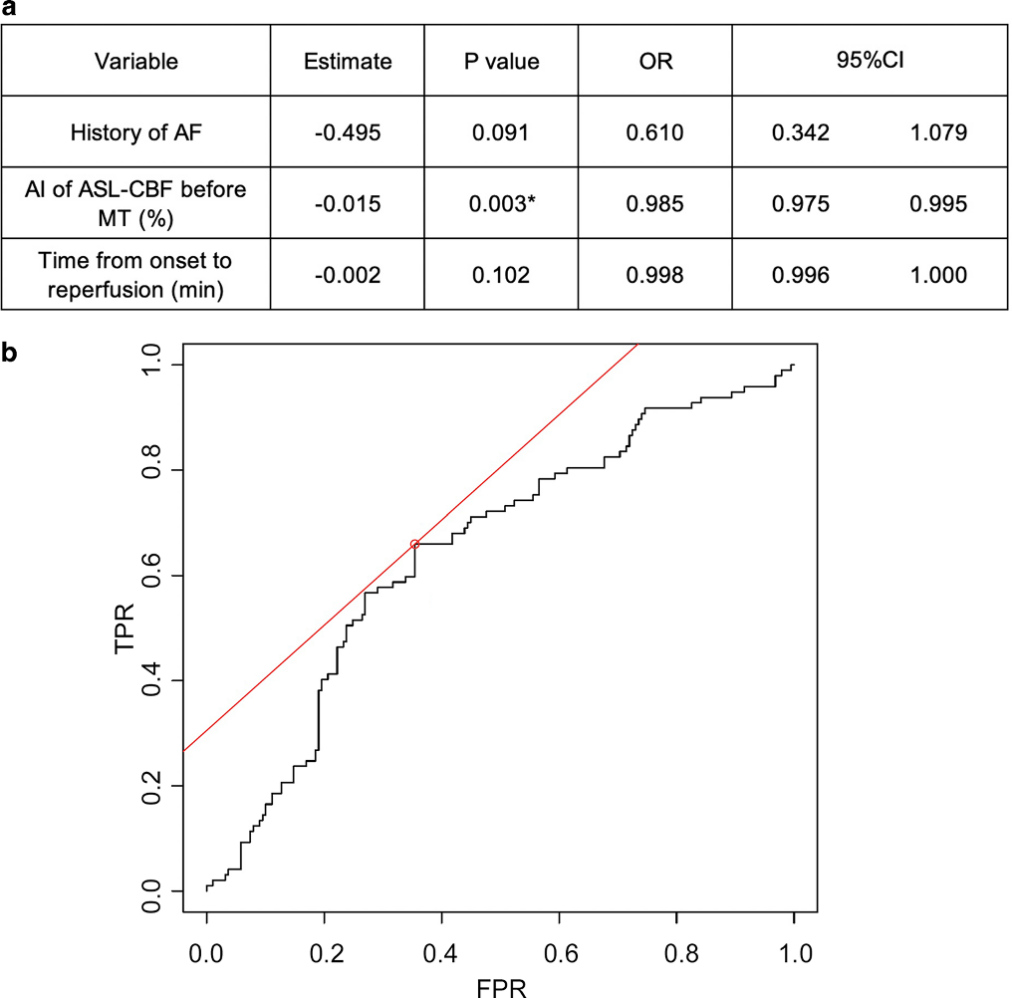
Logistic regression analysis for factors predicting occurrence of CI. The combination of a history of atrial fibrillation, AI of ASL-CBF before MT, and the time from onset to reperfusion could predict the occurrence of CI with the lowest AIC value (AIC = 361) (**a**). AUC of the model = 0.647 (**b**)

Forward and backward stepwise logistic regression analyses with AIC and the previously mentioned factors revealed that the combination of a history of AF, the AI of ASL-CBF before MT, and the time from onset to reperfusion predicted the occurrence of CI after MT, according to the lowest AIC value (AIC = 361, threshold‑1.54, sensitivity 35.4%, specificity 66%) (Fig. 2a). The analysis indicated that if the formula 0.3211 × history of AF +0.0096 × the AI of ASL-CBF before MT (%) +0.0012 × the time from onset to reperfusion (min) yielded a value below 1.0, CI occurred after MT. The AI of ASL-CBF before MT was the only significant predictor (*p* = 0.003, odds ratio, OR = 0.985, 95% confidence interval, CI = 0.975–0.995) (Fig. 2b).

### Receiver Operating Curve (ROC) Analysis

**Fig. 3:**
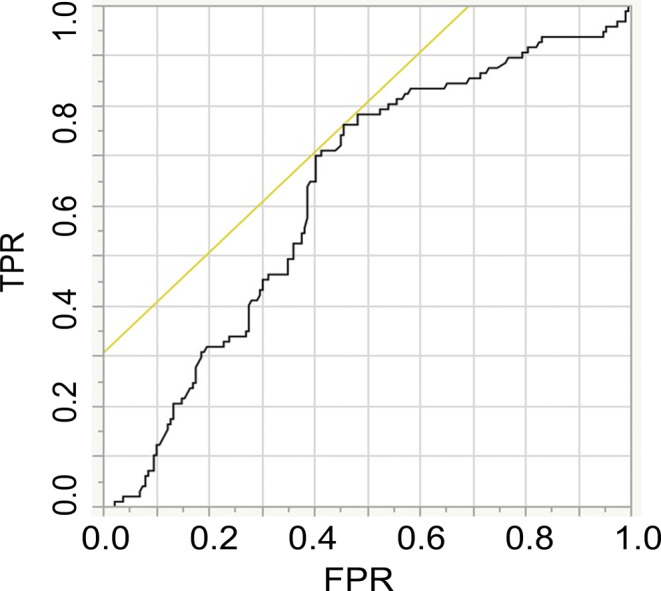
Receiver operating characteristic analysis. The AI of ASL-CBF before MT was significantly correlated to the occurrence of CI after successful MT. The cut-off value for predicting the occurrence of CI was 61.5%

The ROC analysis revealed that the AI of ASL-CBF before MT was significantly related to the occurrence of CI after MT (*p* = 0.0032, AUC = 0.62636). The cut-off value for the prediction of CI after MT was 61.5% (sensitivity 54.5%, specificity 76.3%) (Fig. 3).

### Representative Case

**Fig. 4:**
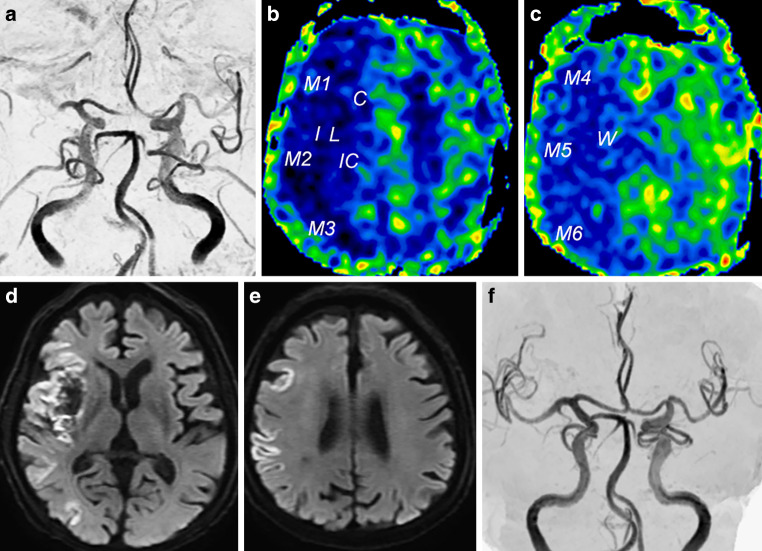
Preoperative MRA illustrated occlusion of the M2 portion of the right MCA (**a**). Preoperative ASL imaging showed that the AI of ASL-CBF was less than 61.5% at the right striatum, insula, M3, M4, M5, and M6 regions of the cerebral cortex. *C* ROI of caudate nucleus, *IC* ROI of internal capsule, *St* ROI of striatum, *M1, M2, M3, M4, M5, and M6* 6 ROIs at the cerebral cortex of the MCA region, *W* ROI of corona radiata (**b**, **c**). Postoperative DWI showed occurrence of CI at the right insula, striatum, M1, M2, M3, M4, and M5 regions of the cerebral cortex (**d**, **e**). Preoperative MRA showed achievement of reperfusion with a TICI score of 3 (**f**)

An 86-year-old man with a history of hypertension and diabetes mellitus presenting with sudden neurological symptoms and an NIHSS score of 15 was transferred to our hospital. Preoperative MRA indicated occlusion of the M2 portion of the right MCA (Fig. 4a). On the preoperative ASL images, the AI of ASL-CBF was below 61.5% at the right striatum, insula, M2, M3, M4, M5, and M6 of the cerebral cortex (Fig. 4b, c). The formula 0.3211 × history of AF + 0.0096 × the AI of ASL-CBF before MT (%) + 0.0012 × the time from onset to reperfusion (min) yielded a value below 1.0 at the right striatum, insula, M2, M3, M4, M5, and M6. Postoperative DWI showed that CI occurred at the right insula, M2, M3, M4, M5, and M6 (Fig. 4d, e). Preoperative MRA showed that reperfusion was achieved, with a TICI score of 3 (Fig. 4f).

## Discussion

The role of ASL in the assessment of ischemic penumbra remains controversial. While ASL overestimates the volume of ischemia more than perfusion MRI with dynamic susceptibility contrast (DSC) [22], the quantitative value of CBF and arterial transit time measured by ASL has been found to show a moderate to high correlation with the value of CBF and mean transit time measured by perfusion CT [23]. The predicted infarct volume defined as that with a mean transit time > 20 s is highly related to that of a CBF measured with ASL of less than 20 ml/100 g [24]. Yoo et al. used the AI of ASL-CBF to examine the outcomes of 51 patients with acute ischemic stroke after MT [18]. They found that the AI of ASL-CBF was significantly larger after compared to before MT. Moreover, the difference between the two values was significantly related to the prognosis of the patients. Their study used both the AI of ASL-CBF before MT and that after MT to predict prognoses. Our study demonstrates that CI can occur even after successful revascularization with MT if the AI of ASL-CBF before MT is below 61.5%, or if the formula 0.3211 × history of AF + 0.0096 × the AI of ASL-CBF before MT (%) + 0.0012 × the time from onset to reperfusion (min) yielded a value below 1.0. To our knowledge, this is the first report indicating that the occurrence of infarction after MT can be predicted only by the AI of ASL-CBF before MT. With those cut-off value, it would be predictable that ischemic brain at each ROI of ASPECT would fall into infarction even after successful MT, leading to create ASL-based ASPECT. Our data will be clinically valuable if this ASL-based ASPECT will prove to be useful alternative to conventional ASPECT.

This study has, however, several limitations. First, our data were derived from patients in whom revascularization with TICI scores of 2B or 3 was achieved; the role of ASL in patients with low revascularization rates by MT remains uncertain. Second, our data were derived from patients who arrived within 8 h after stroke onset. Thus, our results may not apply to those admitted later. Third, there were four patients for whom onset time was defined as the last seen well time. The duration between the last seen well time and the discovery time ranged from 70 min to 360 min in our study. The presence of those patients possibly influenced the results because of the small study group. That is another limitation. Finally, a very limited number of patients were included in our study, which represents one of the biggest limitations of the study; however, the significance of the statistical analysis was ensured. Nevertheless, our results require further confirmation from prospective studies with a larger number of patients.

## Conclusion

Occurrence of CI after successful mechanical thrombectomy for ischemic stroke in the anterior circulation can be expected when the formula 0.3211 × history of AF + 0.0096 × the AI of ASL-CBF before MT (%) + 0.0012 × the time from onset to reperfusion (min) yielded a value below 1.0 or when the AI of ASL-CBF before MT is below 61.5%.
